# Comorbidity and Shared Etiology of ADHD and ASD in Paediatric Populations

**DOI:** 10.1192/j.eurpsy.2026.10545

**Published:** 2026-07-31

**Authors:** P. F. B. Araújo, V. L. de-Melo-Neto, A. F. C. Ximenes, L. V. de Araújo, B. D. A. A. L. T. de Melo, A. B. D. C. Alves, L. C. Carneiro, M. I. O. de Morais, F. O. Barbosa, E. D. F. Correia, D. B. B. Ferreira

**Affiliations:** 1 Psychiatry, Universidade de Ciências da Saúde de Alagoas; 2 Psychiatry, Universidade Federal de Alagoas, Maceió, Brazil

## Abstract

**Introduction:**

Attention-deficit/hyperactivity disorder (ADHD) and autism spectrum disorder (ASD) are increasingly recognised as neurodevelopmental conditions that frequently co-occur in paediatric populations, presenting significant diagnostic challenges. Research indicates a high frequency of this comorbidity, with overlapping symptoms hindering diagnosis and co-occurrence associated with greater functional impairment in children. Since both disorders typically emerge in early development, focusing on paediatric populations is essential to ensure timely identification and intervention. Epidemiological data demonstrate considerable heterogeneity, highlighting the need to explicitly cite meta-analyses when reporting such rates. This highlights the need to clarify comorbidity and shared mechanisms in youth.

**Objectives:**

This systematic review examines current evidence regarding ADHD-ASD comorbidity prevalence, underlying aetiological mechanisms, and neurobiological foundations.

**Methods:**

This literature review was conducted using PubMed databases and Virtual Health Library searches spanning 2020-2025, focusing on ADHD-ASD comorbidity. Search strategies employed “ADHD”, “autism spectrum disorder”, and “comorbidity” using Boolean operator AND. Studies met inclusion criteria if they: (1) included paediatric samples, (2) were published in English or Portuguese, and (3) addressed prevalence, aetiology, neurobiology, or therapeutic interventions. Exclusion criteria comprised case reports, editorials, and studies lacking mental health focus.

**Results:**

Autism spectrum disorder and ADHD represent neurodevelopmental conditions with prevalence rates of 1.5-2% and 5-9%, respectively, according to (Hatch et al. Psychol Sch 2022; 60:295-311). Comorbidity rates vary: 53-78% of children with ASD exhibit ADHD symptomatology, whilst 21% with ADHD display autistic traits. Both conditions demonstrate substantial heritability: ADHD shows 70-80% whilst ASD ranges 37-90%, with approximately 40% genetic overlap indicating shared mechanisms. Neuroimaging reveals overlapping dysfunction across attention networks and executive control systems. Children with both conditions experience greater functional impairments, higher anxiety, increased academic difficulties, and reduced therapeutic responsiveness. Environmental risk factors include maternal stress and toxin exposures. Effective approaches require comprehensive multimodal interventions combining behavioural and pharmacological therapies.

**Conclusions:**

The substantial phenotypic and genetic overlap between ADHD and ASD necessitates integrated diagnostic frameworks recognising shared presentations and mechanisms. Given significant genetic convergence and overlapping neurobiological pathways, these conditions may be conceptualised as related spectrum disorders requiring specialised therapeutic approach. Comprehensive assessment and multimodal interventions remain essential for optimal outcomes.

**Disclosure of Interest:**

None Declared

